# Recurrence of moderate to severe ulcerative colitis after fecal microbiota transplantation treatment and the efficacy of re-FMT: a case series

**DOI:** 10.1186/s12876-020-01548-w

**Published:** 2020-11-26

**Authors:** Xiao-Fei Dang, Qing-Xi Wang, Zhao Yin, Lin Sun, Wei-Hua Yang

**Affiliations:** 1 Department of Clinical Microbiology, Medical Research & Laboratory Diagnostic Center, Jinan Central Hospital Affiliated to Shandong First Medical University, 105 Jiefang Road, Jinan, Shandong China; 2 Gastroenterology, Jinan Central Hospital Affiliated to Shandong First Medical University, 105 Jiefang Road, Jinan, China

**Keywords:** Fecal microbiota transplantation, Ulcerative colitis, Remission, Recurrence

## Abstract

**Background:**

Ulcerative colitis (UC) is a chronic inflammatory bowel disease (IBD), the pathogenesis of which is complicated, and it is difficult to treat. In recent years, the emerging fecal microbiota transplantation (FMT) has shown good effects in UC treatment and is therefore accepted by increasing numbers of patients. Our hospital has carried out FMT since 2017, and has achieved good results in UC treatment. We have found in our clinical work that the efficacy of re-FMT after recurrence decreased. This is difference from reported literatures. In order to attract clinical attention, here we selected typical cases for analysis.

**Methods:**

Among all UC patients who received FMT in our hospital, 12 patients with moderate to severe UC were selected. They all received multiple FMT and were followed up for 52 weeks. Besides, none of them had other underlying diseases. Colonoscopy images of patients were presentated, SCCAI and UCDAI were used assess the effect of FMT.

**Results:**

On the whole, FMT has a significant effect on moderate to severe UC. Of the 12 patients, 11 (91.7%) achieved a clinical response, 9 (75.0%) achieved clinical remission, and only one patient did not respond to FMT treatment. However, 6 patients relapsed within 52 weeks after remission, with a recurrence rate of 54.5%. Four of the six relapsed patients received FMT again, but the efficacy of FMT after relapse was significantly lower than that of the initial FMT. Fortunately, compared to before the initial FMT treatment, the severity of the disease after relapse was significantly reduced.

**Conclusion:**

FMT has a good effect on the relief of moderate to severe UC. However, the effect of FMT treatment after relapse is reduced. For patients who relapse after remission, the efficacy of FMT reapplication requires more experiments to verify.

## Background

Ulcerative colitis (UC) is an inflammatory bowel disease (IBD) that causes long-lasting inflammation and ulcers (sores) in the digestive tract. The primary goals of treatment are to induce and maintain clinical and endoscopic remission [1]. The likely pathogenesis of UC involves changes in the composition of the gut microbiota, known as “dysbiosis″, which can cause activation of the mucosal immune system, leading to chronic inflammation of the mucosa [2]. Therefore, fecal microbiota transplantation (FMT), which can remodel the microbiota balance of the flora, has become an effective method for the treatment of mild to moderately active UC [3].

The purpose of FMT is to restore the intestinal microbiota balance by the transfer of microbiota isolated from “healthy″ donor feces to the recipient’s intestine [4]. FMT reduces intestinal permeability by increasing the production of short-chain fatty acids (especially butyrate), thereby reducing the severity of UC. This, in turn, helps to maintain the integrity of the epithelial barrier and it can inhibit Th1 differentiation, T cell activity, leukocyte adhesion, and inflammatory factors from restoring immune dysbiosis [5]. It was reported that five patients with moderate-to-severe active UC were followed up for 3 months after FMT, and for most of them, their gut microbiota was changed by FMT in the first 3 days, but then reverted to the initial state in 1 to 4 weeks [6]. It has also been reported that inflammatory bowel disease (IBD) patients require several FMTs to stabilize the altered intestinal microbiota [7].

Since 2017, our hospital implemented FMT for UC patients. In order to present the clinical outcomes and recurrence rate of patients with moderate to severe UC who received multiple FMT treatments, we summarized the UC patients who underwent multiple FMT treatments and completed the retrospective study after long-term follow-up in Jinan Central Hospital.

## Methods

### Study population

From 2017 to 2020, a total of 202 UC patients underwent FMT treatment in our hospital. We sorted out the cases and found that 12 patients with moderate to severe UC received multiple FMT, and there were no other underlying diseases except for UC.

The study cohort included all the 12 patients. Relevant demographic and clinical data were retrieved from patient files and electronic records. This retrospective study has obtained the patient’s informed consent and approved by the ethics review committee of Jinan Central Hospital.

### FMT treatment

All 12 patients received standardized FMT in our hospital. The process and standards are as follows:

1. Donor status: Possible stool donors were screened out through a questionnaire. Then undergo strict laboratory inspections, and the qualified individuals were selected out as the donor. Additional file 1 includes a complete overview of the donor screening. Donors were not allowed to use antibiotics for 12 weeks before screening. Patients were infused with fecal extracts from only one donor at a time.
2. Preparation of fecal microbiota: The donors provided the feces within 15 min after defecation. Fecal microbiota were extracted through filtration, centrifugation, and washing. Finally, 15 ml bacterial pellet was mixed with 75 ml physiological saline, which was the fecal microbiota provided to patients clinically. If the fecal extract was not used within 3 h, it could be stored at − 80 °C and thawed before infusion.
3. Fecal bacteria input: 90 mL of fecal microbiota was injected directly into the terminal ileum or cecum through the colonoscope.

### Efficacy evaluation

At follow-up, the Ulcerative Colitis Disease Activity Index (UCDAI) [8] was used to evaluate the severity of the patients’ condition. At each visit, patients’ defecation frequency and bleeding, gastrointestinal symptoms, adverse events, and drug changes were evaluated. Adverse events were evaluated using the Common Terminology Criteria for Adverse Events (CTCAE) version 4.03. All of the patients maintained a colonoscopy record and we reviewed it to monitor their treatment response (The colonoscopy record template was in Additional file 2). At the time points when no colonoscopy was performed, their quality of life was evaluated using the Simple Clinical Colitis Activity Index (SCCAI) [9] at 1, 6, 12, and 52 weeks after the first FMT. A clinical response was defined as a ≥ 3 improvement in the SCCAI or UCDAI score. A clinical remission was defined as a full SCCAI, or a UCDAI score of 2 points or lower.

## Results

### UC duration and details of patients

The study population included 12 patients with moderate to severe UC. There were 8 (66%) men and 4 (34%) women, with a median age of 50.5 years (range: 41–65 years). Five (41.7%) patients were diagnosed with moderate UC, and seven (58.3%) patients were diagnosed with severe UC. The history of present illness showed all patients with active UC for a median duration of 4 mo (range: 1–12 mo).

About UC duration, that of 10 patients was over 1 year, and all patients have a median duration of 3.5 years (range: 3 mo to 20 years). Table 1 summarizes the baseline characteristics and laboratory data of the 12 patients.

**Table 1:** Baseline characteristics of patients

Patient	Age	Sex	Disease type	Previous treatment	Stool routine	ESR (mm/h)	CRP (mg/l)	Stool frequency	Rectal bleeding	UC duration
Patient 1	65	Female	Moderate	5-ASA	(+)	115	20	8–10	None	2 y
Patient 2	58	Female	Severe	Glucocorticoid;5-ASA	(+)	20	1.1	5–20	Obvious blood	2 y
Patient 3	46	Male	Severe	5-ASA	(+)	25	8.6	10	Mostly blood	3 mo
Patient 4	52	Female	Moderate	5-ASA	(+)	22	1	4–5	Obvious blood	5 y
Patient 5	41	Male	Moderate	Traditional Chinese medicine	(+)	7	1.2	3–4	Obvious blood	1 y
Patient 6	49	Male	Severe	Glucocorticoid; 5-ASA	(+)	18	7.63	9–10	Obvious blood	10+ y
Patient 7	53	Female	Severe	Glucocorticoid; 5-ASA	(+)	36	9.8	8–10	Mostly blood	9 y
Patient 8	49	Male	Moderate	5-ASA	(+)	6	–	4–5	Obvious blood	4 mo
Patient 9	43	Male	Severe	5-ASA	(+)	21	12.6	4–5	Mostly blood	10 + y
Patient 10	52	Male	Severe	Glucocorticoid;5-ASA	(+)	3.11	<0.1	>10	Obvious blood	6 y
Patient 11	48	Male	Moderate	5-ASA	(+)	37	45.7	6–8	Obvious blood	1 y
Patient 12	58	Female	Severe	Glucocorticoid;5-ASA	(+)	48	36.1	>10	Mostly blood	20 y

Exp. arm: *y* year, *mo* month

### Clinical remission

We reviewed the 52-week follow-up records and analy of 12 patients and found three outcomes: no recurrence after remission (Group A), recurrence after remission (Group B), and no remission (Group C). Among them, we found that effect of FMT is reduced after recurrence.

In the 12 patients who underwent repeated FMT for moderate-and-severe UC, 11 patients (Group A and Group B) achieved a clinical response, and 5 patients were still in clinical remission at 52 weeks of follow-up. However, six patients (Group A) relapsed within 52 weeks after achieving a clinical remission, and only 1 patient (Group C) did not respond to FMT. We found that patients who had a remission after FMT and did not relapse within 52 weeks had a median disease duration of only 2 years (range: 3 mo to 5 years). In contrast, patients who relapsed after remission had a median disease duration of 7.5 years (range: 4 mo to 20 years).

### Effect of re-FMT

Four patients (Group B) underwent an additional FMT after relapse, but the re-application of FMT did not achieve the desired effect. In addition, all patients had good compliance and no adverse events were observed after FMT during the follow-up period (Table 2).

**Table 2:** Outcome of FMT and adverse events

	Patient	UCDAI before FMT	SCCAI before FMT	Clinical response	Clinical remission	Relapse time	SCCAI at week 52	AE
GroupA	Patient 1	8	9	yes	yes	–	2	No
	Patient 2	9	8	yes	yes	–	1	No
	Patient 3	10	10	yes	yes	–	2	No
	Patient 4	7	8	yes	yes	–	2	No
	Patient 5	6	8	yes	yes	–	2	No
GroupB	Patient 6	10	11	yes	yes	52 w	9	No
	Patient 7	12	12	yes	no	8 w	8	No
	Patient 8	8	9	yes	yes	48 w	8	No
	Patient 9	11	10	yes	no	26 w	9	No
	Patient 10	9	10	yes	yes	13 w	9	No
	Patient 11	8	8	yes	yes	28 w	8	No
GroupC	Patient 12	12	12	no	no	–	11	No

Exp. arm: *UCDAI* Ulcerative Colitis Disease Activity, *AE* Adverse event; *w* weeks

### Cases in detail

**Fig. 1:**
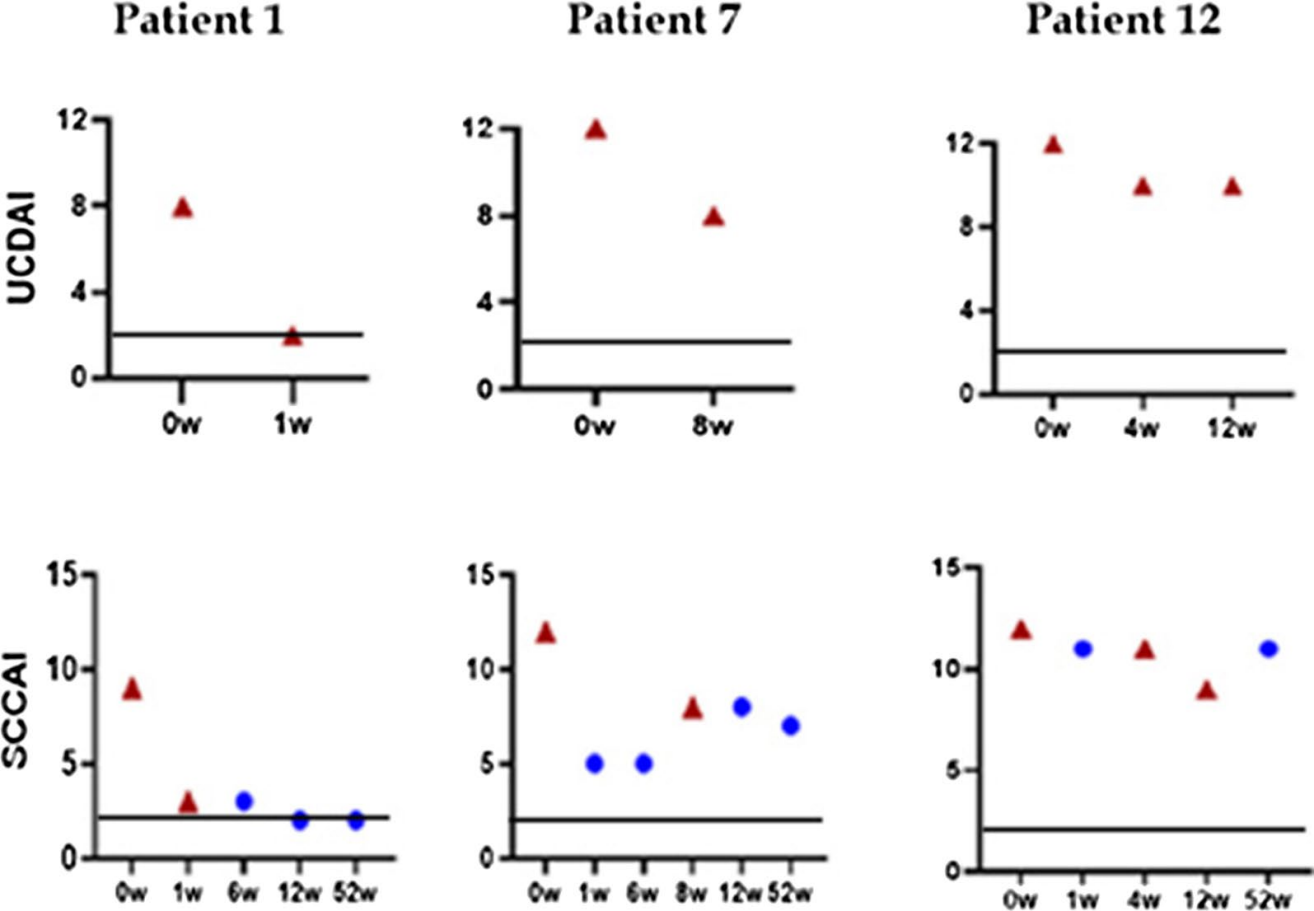
Clinical assessment summary chart. Exp. arm: SCCA: Simple Clinical Colitis Activity; UCDAI: Ulcerative Colitis Disease Activity; triangle indicates that the FMT was performed during this assessment; circle indicates that the FMT was not performed

We present the detailed cases of one patient of each group in the following. Figure 1 is a summary of their UCDAI and SCCAI, which clearly illustrates the changes in the patients’ conditions.

**Fig. 2:**
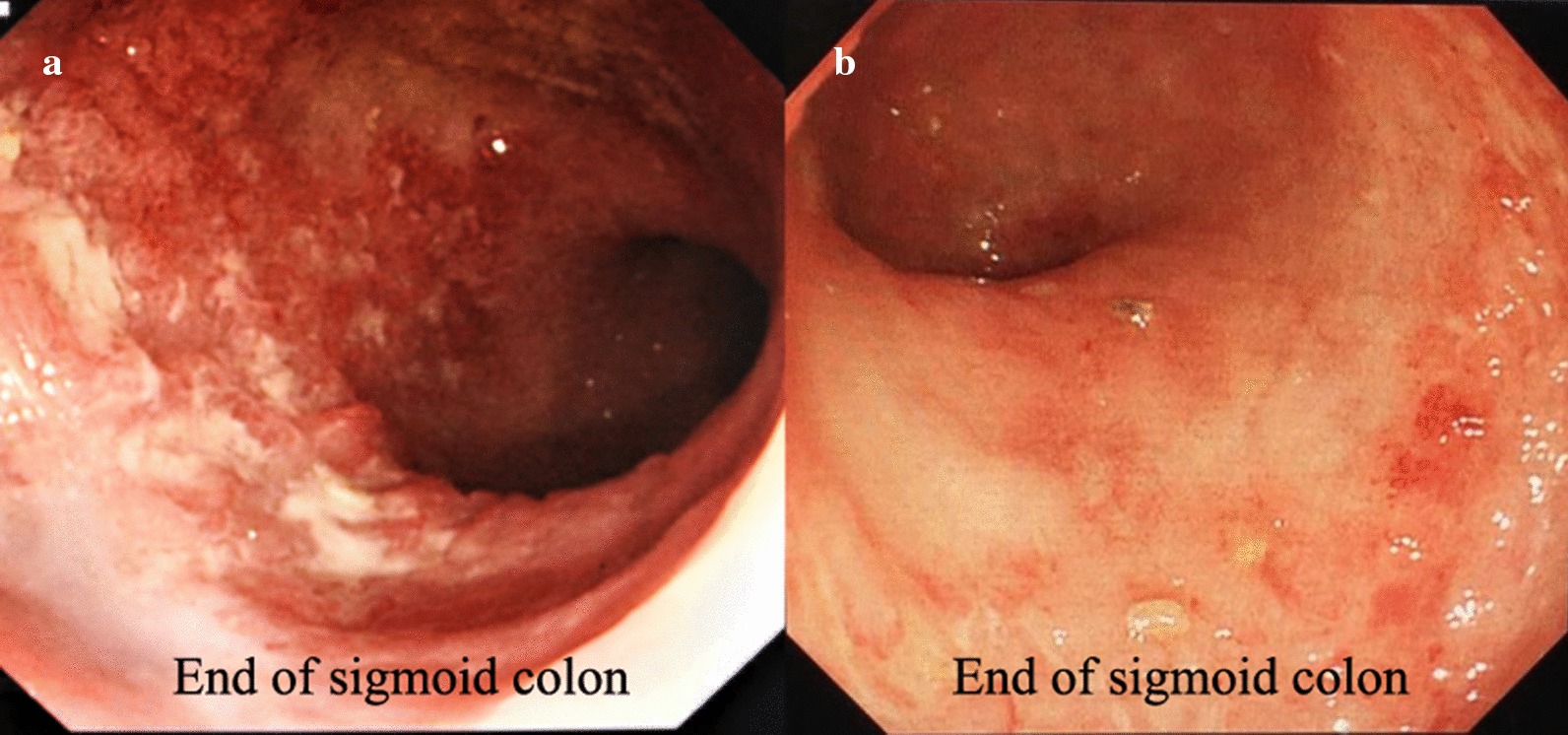
Comparison of two colonoscopies of patient 1. **a**: 2018.01.02 **b**: 2018.01.10

Patient 1 was a 65-year-old woman. In January 2018, she was admitted to the hospital with repeated diarrhea for 4 months. ESR 115 mm/h, CRP 20 mg/l. Colonoscopy on January 3, 2018, revealed that the patient’s colonic mucosa displayed hyperemia, erosion, and multiple superficial ulcers, with the surface being covered with purulent secretions. The rectal mucosa was smooth, and her UCDAI score was 8. FMT was performed with a colonoscope (Fig. 2a). At week 2, the patient was treated with FMT by colonoscopy again. On January 10, 2018, her UCDAI decreased to 2 points. ESR 16 mm/h, CRP 1.0 mg/l. The intestinal mucosa seen by colonoscopy is shown in Fig. 2b. The patient took 1 g of mesalazine granules orally 3 times a day and did not relapse until week 52.

**Fig. 3:**
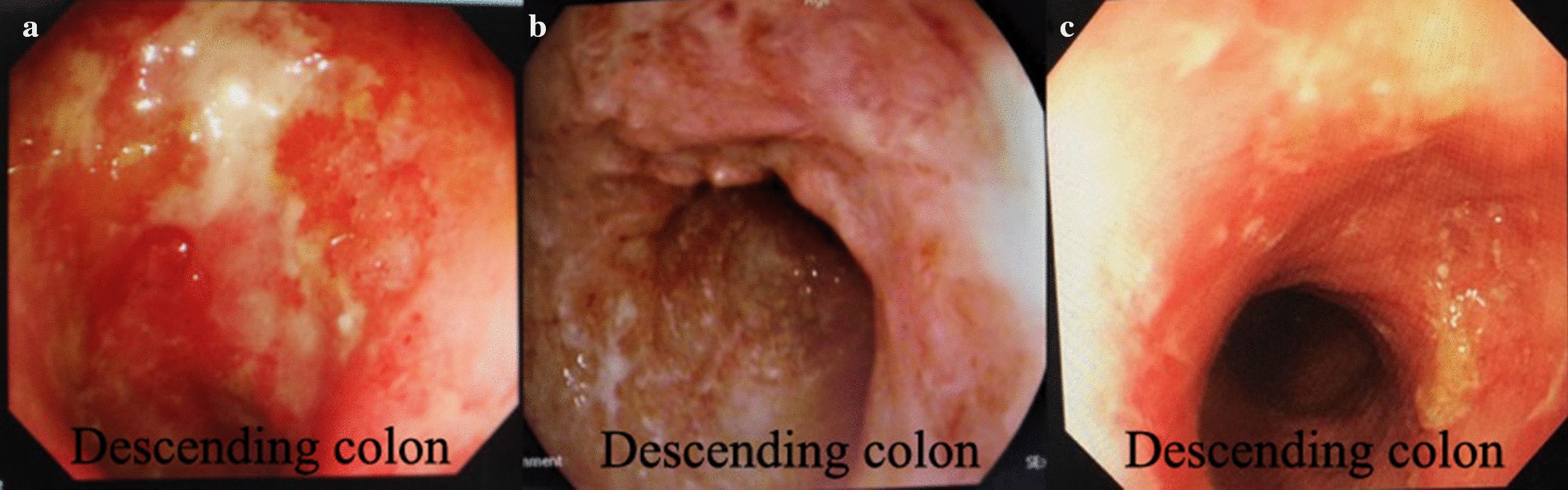
Comparison of two colonoscopies of patient 7. **a**: 2019.01.11 **b**: 2019.03.13

Patient 7 was a 53-year-old Chinese woman who had suffered from UC for 9 years. Colonoscopy on January 11, 2019, revealed diffuse hyperemia and erosion of the entire colon and rectum, multiple superficial ulcers, and pseudo polyps. Her UCDAI was 12. ESR 36 mm/h, CRP 9.8 mg/l. A titanium clip was used to fix the colonic transendoscopic enteral tubing (TET) in the ileocecal region (Fig. 3a). A total of 90 ml of bacterial material was infused on the 1st, 3rd, and 5th day using the TET tube left in the anus. In week 2, the patient’s stool frequency declined, with a decrease of the pus and blood in the stool. ESR 28 mm/h, CRP 7.66 mg/l. In week 8, however, the patient’s pus and blood increased again, and the stool was not formed. A TET was placed again by colonoscope on March 13, 2019. The mucosal condition of the colon is shown in Fig. 3b. Her UCDAI was 8. The patient underwent 3 treatments FMT once again via the TET. However, the desired effect after FMT treatment was not achieved, and the patient resumed steroid therapy.

**Fig. 4:**
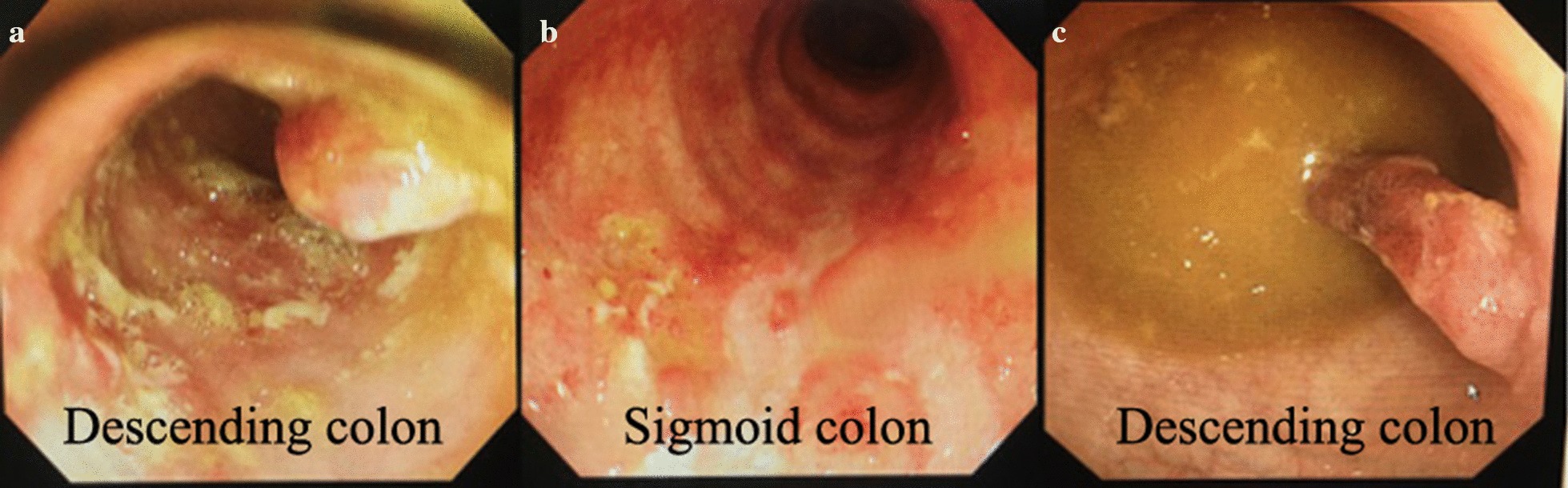
Comparison of three colonoscopies of patient 12. **a**: 2017.10.10 **b**: 2017.11.10 **c**: 2018.01.09

Patient 12 was a 58-year-old woman who was admitted to the hospital in October 2017 because of repeated mucopurulent stool lasting for more than 20 years. Most of the patient’s stool was bloody, and oral glucocorticoids and mesalazine could not alleviate these symptoms. The test results showed ESR 48 ml/h and CRP 36.1 mg/l. A colonoscopy revealed mucosal congestion, surface ulceration, and purulent secretions in the entire colon and rectum (Fig. 4a). Her UCDAI was 12. Subsequently, the patient underwent 2 FMTs through colonoscopy, and no clinical results were obtained. ESR 32 ml/h and CRP 22.1 mg/l.

## Discussion and conclusions

The etiology and pathogenesis of UC are complex. Many studies support the view that it is caused by an imbalance between the intestinal flora and mucosal immunity, which leads to excessive inflammation [10, 11]. The composition diversity of the intestinal flora of UC patients is lower than that of general population, and the XI and Va bacteria of Clostridium are reduced [12, 13]. In patients with IBD, a structural and functional imbalance of the intestinal flora has been reported many times [14–16]. FMT has become a new method to change the intestinal microbiome and has been successfully used to treat refractory Clostridium infection (CDI) [17]. A published systematic review conducted by Costello et al. [18] conducted a meta-analysis of 14 cohort studies and 4 RCTs to prove the effectiveness of FMT in the treatment of active UC, including 308 patients with UC who received FMT. In these meta-analyses of RCTs, it was reported that FMT effectively treated UC with a clinical remission rate of 28% (39/140) in patients treated with FMT, compared with 9% (13/137) in patients treated with the placebo. Furthermore, clinical response was achieved in 49% (69/140) of patients treated with FMT compared with 28% (38/137) of patients treated with the placebo. In the 14 cohort studies, 24% (39/168) of patients treated with FMT achieved clinical remission.

Through our search in the electronic database, only two studies conducted long-term follow-up of UC patients treated with FMT [19, 20]. A total of 57 patients were involved, and 40 patients maintained remission at 48 weeks. However, none of the studies evaluated the effect of FMT in patients with UC after relapse.

Continuous FMT could promote intestinal regulation, alter the host immune status and the intestinal barrier, and improve the clinical response to drugs [21]. Increasing the application frequency and prolonging the treatment time of FMT applications can achieve a better clinical remission [22]. One-time or short-term repeated FMT may result in failure of the clinical treatment. For the long-term and the sustainable change of gut microorganisms, repeated treatments over a few weeks are necessary. Zhang’s team studied the therapeutic effects of FMT on steroid-dependent UC. After multiple FMT applications, more than half of the patients achieved clinical improvements [21].

In our retrospective study, FMT had a significant effect on moderate to severe UC. Among the 12 patients, 11 patients achieved a clinical response, and 5 patients were still in clinical remission at 52 weeks of follow-up, although six patients relapsed within 52 weeks after reaching a clinical remission, while only 1 patient did not respond to FMT.

The three different outcomes observed in this report can be primarily attributed to the differences in the duration of the disease and severity of the disease. We found that the median disease duration of patients who did not relapse within 52 weeks after FMT treatment was 2 years (range: 3 mo to 5 y). In contrast, the median disease duration of patients who relapsed after remission was 7.5 years (range: 4 mo to 20 years). In addition, the overall status of the 6 recurrent patients and the patient without any effect were more severe than the 5 patients who achieved a complete remission (including CRP, ESR, duration of illness, etc.).In addition to the above research, many studies are currently examining the effects of FMT on chronic diseases, and it has been found that super donors may exist; that is, feces of a particular donor are more likely to succeed in FMT than that of other donors [23]. A rigorous screening of donors in our hospital is conducted.

We have achieved better clinical efficacy than reported in the literatures, because the quality of fecal bacteria isvital to the efficacy of FMT. During preparation, the exposure time of fecal microbiota in the air should be reduced to ensure the survival of bacteria. The time of the fecal microbiota preparation in our hospital is about 2 h, which is shorter than the 6 to 12 h reported in other studies [24]. This may be one of the reasons why we had such a high clinical response (11/12) and a prolonged clinical remission (9/12) over 1 year.

In summary, our study evaluated the efficacy and safety of multiple consecutive FMTs in patients with moderate-to-severe UC. We conducted a 52 weeks follow-up, and found after a relapse, the effect of FMT treatment was not as good as that of FMT before a relapse, but the final result was still much better than before the first FMT. Like other treatments, relapses after FMT are more likely to occur in very severe patients, and the efficacy of FMT will decrease after a relapse.

## Supplementary Information

**Additional file 1**. Principles of Donor Selection.

**Additional file 2**. Colonoscopy record template.

## Data Availability

The datasets generated during and/or analysed during the current study are available from the corresponding author on reasonable request.

## Abbreviations

FMT: Fecal microbiota transplantation
IBD: Inflammatory bowel disease
UC: Ulcerative colitis
ESR: Erythrocyte Sedimentation Rate
CRP: C-reactive protein
y: Year
mo: Month
UCDAI: Ulcerative Colitis Disease Activity Index
SCCAI: Simple Clinical Colitis Activity Index
AE: Adverse event
w: Weeks
TET: Colonic transendoscopic enteral tubing
