# Supplementary material for: Recurrence of moderate to severe ulcerative colitis after fecal microbiota transplantation treatment and the efficacy of re-FMT: a case series
Source: BMC Gastroenterol. 2020 Nov 26;20:401. doi: 10.1186/s12876-020-01548-w (PMC7691068; doi:10.1186/s12876-020-01548-w)
Supplement: Supplementary file 1 — Additional file 1. Principles of Donor Selection. [file 12876_2020_1548_MOESM1_ESM.docx]

**Questionnaire:**

Do you have infectious diseases such as hepatitis, syphilis, HIV, schistosomiasis

Have you experienced acute enteritis within 6 months

Have you taken antibiotics in 3 months

Have you had blood transfusions within 6 months

Have you been to an infectious disease epidemic area within 6 months

Have you used hormones and immunosuppressants in 6 months

Have you experienced constipation or bloating within 3 months

Have you had more than two colds in 3 months

Have you used cathartic or intestinal lavage within 3 months

Have you been diagnosed with inflammatory bowel disease, peptic ulcers and gastritis

Have you ever had a gastrointestinal polyposis or a tumor

Do you have recurrent allergies or typical skin disorders

Do you have an autoimmune disease

Do you have rhinitis, asthma, chronic obstructive pulmonary disease

Whether you and your immediate family members have a mental or mental illness

Whether you and your immediate family members have endocrine system diseases (such as diabetes)

Whether you and your immediate family members have high blood pressure

Do you have regular bowel movements?

Do you have sex within 6 months, and if so, does your partner have the above problems?

**Laboratory inspection:**

liver function

Renal function

Blood ion analysis

Blood biochemistry

Blood routine

Tumor marker

Infectious marker

90 allergen tests

Helicobacter pylori antibody

Mycobacterium tuberculosis antibody test

DNA detection of various pathogens

Urine routine

Stool routine

Stool culture
