# Supplementary figures and images for: Recurrence of moderate to severe ulcerative colitis after fecal microbiota transplantation treatment and the efficacy of re-FMT: a case series
Source: BMC Gastroenterol. 2020 Nov 26;20:401. doi: 10.1186/s12876-020-01548-w (PMC7691068; doi:10.1186/s12876-020-01548-w)

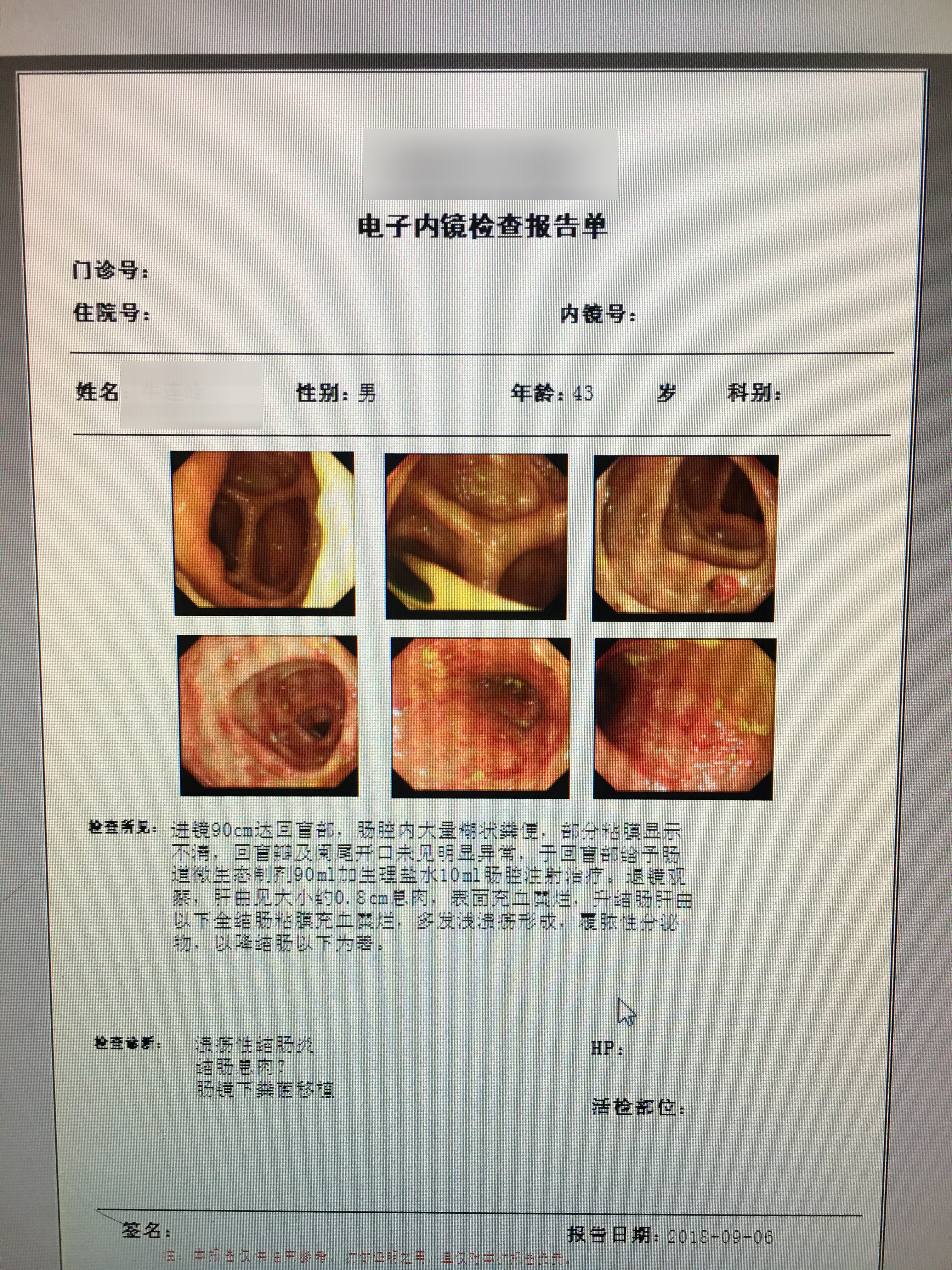

Supplement: Supplementary file 2 — Additional file 2. Colonoscopy record template. [file 12876_2020_1548_MOESM2_ESM.tif]
